# Capacity of the CCC-2 to Discriminate ASD from Other Neurodevelopmental Disorders

**DOI:** 10.3390/children8080640

**Published:** 2021-07-27

**Authors:** Alba de la Torre Carril, Montserrat Durán-Bouza, Miguel Pérez-Pereira

**Affiliations:** 1 Department of Psychology, Faculty of Educational Sciences, University of A Coruña, 15008 A Coruña, Spain; alba.delatorrecarril@outlook.com (A.d.l.T.C.); montserrat.duran.bouza@udc.es (M.D.-B.); 2 Department of Developmental and Educational Psychology, Faculty of Psychology, University of Santiago de Compostela, 15782 Santiago de Compostela, Spain

**Keywords:** CCC-2, communicative impairments, pragmatic disorders, autism spectrum disorder, neurodevelopmental disorders

## Abstract

The Children’s Communication Checklist (CCC-2) has demonstrated its usefulness as an instrument to assess discrepancies between the use of structural dimensions of language and the pragmatic and sociointeractive uses of language. The aims of the present paper are: (1) to test the capacity of the Galician adaptation of the CCC-2 to discriminate the linguistic profiles of children with different disorders and (2) to test whether the capacity of the CCC-2 to discriminate the linguistic abilities of children with different disorders is the same at different ages: earlier development and later development. The sample is of 117 children previously diagnosed with different disorders: autism spectrum disorder (ASD), developmental language disorder (DLD), attention deficit with hyperactivity disorder (ADHD), Down syndrome children (DS) and typically developing children (TD). The children were divided into two different age groups: from 4 to 6 and from 7 to 16 years of age. The results indicate that the Galician CCC-2 (1) accurately identified children with and without communicative impairments, (2) distinguished between profiles with a predominance of pragmatic (ASD and ADHD) and structural disorders (DS and DLD) and (3) distinguished between different profiles of pragmatic impairment. The CCC-2 equally identified these profiles at both earlier and later ages. The Galician CCC-2 seems to be a useful instrument for differentiating among different clinical groups and for assessing pragmatic disorders from an early age, which can be valuable for planning early intervention.

## 1. Introduction

The main aim of the present paper is to verify the ability of the Galician version of the Children’s Communication Checklist 2nd edition (CCC-2) [1] to differentiate the linguistic profiles of children with autism spectrum disorder from those with other neurodevelopmental disorders.

The CCC-2 inventory [2] was designed to explore structural and pragmatic aspects of language separately and thus be able to detect difficulties that differentially affect pragmatic or structural aspects. The CCC-2 must be completed by an adult who knows the child well; that is why it is aimed at parents, teachers and/or therapists. Although its application is fast, the number of items is sufficient to obtain different information within each of the explored areas [3].

Form, content and use of language are the essential components of human communication. Form (phonology, morphology and syntax) and content (semantics) represent structural skills, while the appropriate use of language in different situations or social contexts has to do with pragmatic skills [4]. Language disorders are related to difficulties in one or more of these skills, depending on age, intellectual level and coexisting difficulties in other developmental domains [5].

Traditionally, pragmatic skills have received less attention than structural skills and there is a lack of agreement regarding a precise definition of them [6,7]. Pragmatics, as indicated above, refers to the ability to express intentions and use language appropriately in interactions with other people and to correctly interpret language in social contexts or in communicative exchanges [8]. The American Speech Language and Hearing Association [9] considers that pragmatic skills include: (1) the ability to perform different speech acts, (2) the ability to modify language depending on the needs of the interlocutor or the situation and (3) the ability to follow the rules of conversation and narration. At present, however, there are numerous studies that consider that the assessment of pragmatic skills is of special importance to differentiate between different clinical groups [10,11,12,13]. In this regard, it is important to highlight that pragmatic disorders are produced both by structural difficulties and by problems of social communication [14]. That is the reason why tests are needed to accurately analyze the various components of language.

Up until now, psychometric tests have been the main instruments used in the evaluation of children with language disorders. A second type of instrument used to identify pragmatic disorders are inventories or questionnaires addressed to parents and/or caregivers. These instruments became relevant because of the deficiency found in the assessment of pragmatic skills from a standardized test and because of the difficulty in observing pragmatic performance in clinical environments. At present, inventories are considered a tool of choice for the evaluation of pragmatics, being more effective than formal tests to detect communication problems [15,16]. They make it possible to obtain representative information on the habitual behavior of the child in different contexts, also allowing access to information that is difficult to observe directly in a clinical context, with reduced time costs. In addition, parents are reinforced by being considered a key element in the process of obtaining information, promoting an active role that will be fundamental for the intervention [17].

Screening tools play a fundamental role in early detection and diagnosis of language disorders associated with neurodevelopmental disorders, including ASD. For this reason, knowing the linguistic profile of the different disorders is essential for the interpretation of the information collected with these instruments. As indicated at the beginning of the section, the objective of this paper is to verify whether the Galician-language version of the CCC-2 [1] is a useful tool to identify children with ASD as opposed to other disorders with linguistic difficulties associated. Due to this, we will compare profiles with a predominance of pragmatic difficulties Autism Spectrum Disorder (ASD) and Attention Deficit with Hyperactivity Disorder (ADHD) and profiles with a predominance of structural difficulties Down syndrome (DS) and Developmental Language Disorder (DLD).

Autism spectrum disorder is characterized by disorders of social interaction and communication and restricted, repetitive and stereotyped interests and activities [18]. Its prevalence in childhood is 1% with these children presenting heterogeneous profiles [19]. At the linguistic level, in the 1990s it was indicated that 50% of children with ASD did not acquire oral language; at present, however, this figure has been reduced to 20–25% [20]. Among those who present oral language, the profile is also varied, showing different degrees of phonological, morphological, syntactic and semantic disorders [21,22,23,24,25]. The pragmatic involvement, however, is universal [13,26].

The following description of the linguistic profile of these children refers to those with verbal autism without significant cognitive limitation. In this type of autism, phonological and morphosyntactic skills are relatively preserved, although at early ages they may show problems [23,24].

Although linguistic deficits in structural aspects of language are not always present in ASD, most pragmatic skills are severely impaired with the ASD profile being variable [13]. Children with ASD hardly initiate conversations and tend to use simple question–answer sequences in conversations, sometimes with irrelevant content [25]. They have difficulties in maintaining the topic and in introducing new topics and shifting them. Children with ASD introduce new topics abruptly and according to their own interests. They present deficiencies in making inferences on the needs of the interlocutor and in maintaining a cooperative dialogue, due to their difficulties in the theory of mind [23,27,28]. Children with ASD are also unable to use and understand gestures, intonation, gaze and, in general, body language [13]. Their speech acts are focused on the instrumental function, so they rarely make comments. References to their inner world are scarce and they show difficulties in understanding the intentions of others. On the other hand, they present difficulties in understanding ironies, metaphors, jokes, idiomatic phrases and indirect requests and problems in inferring ambiguous meanings and, above all, mental states. These difficulties are related to their incapability for relying on the context [23,29]. They present referential communication problems in narratives, including irrelevant content and a lack of coherence. Many times, their narratives hardly contain data about the motivations of the characters and the causal relationships between events [30,31].

Regarding attention deficit hyperactivity disorder, it is a neurodevelopmental disorder characterized by the presence of a set of symptoms derived from inattention, hyperactivity and/or impulsivity [32]. Studies suggest that around a third of children with ADHD also have a developmental language disorder, which would make it difficult to determine whether the language problems present in ADHD are due to this diagnosis or to its comorbidities [33]. A meta-analysis carried out by Korrel et al. [34], however, suggests that language difficulties are present even when there are no comorbid language disorders. Huntchinson et al. [35] linked these difficulties to impairments of executive functions and, above all, of working memory.

Most studies that try to establish the linguistic profile of ADHD report difficulties at a pragmatic level and a smaller number of structural difficulties [34,36,37,38], which have not always been found [39,40]. These studies tend to place communication difficulties at the level of high-functioning autism, although of a lower severity.

Pragmatic impairments affect social performance. Children with ADHD show difficulty in waiting for turns and following instructions, interrupting others, answering questions before they are asked and presenting excessive speech during spontaneous conversation. They do, however, show restricted speech in response to direct questions. They tend to impose the topic of conversation, provide tangential information, do not respond to questions or requests and provide limited feedback [41,42,43]. Children with ADHD have difficulties in interpreting non-verbal and emotional cues; they attend to irrelevant cues and have difficulties understanding irony and using forms of courtesy [44]. Executive functioning difficulties could explain some of the symptoms on the pragmatic level. Thus, the inability to maintain attention would be related to the problems in making inferences, while the planning difficulties would be reflected in narrative discourse: a lack of coherence and cohesion, brief elaborations with little planning and causal guidance, frequent repetitions and ambiguous references [32,38,45]. At the comprehensive level, they tend to retain details and lose the overall idea [46].

Social communication disorder (formerly pragmatic language impairment) stands out among the pragmatic language disorders. However, as it is not the object of study in the present work, its characteristics will not be described.

In contrast, other disorders such as Down syndrome and developmental language disorder present a predominance of structural difficulties.

In the case of children with DS, phonology and syntax are the most affected areas [47,48]. Phonological errors have to do, above all, with the omission of syllables, simplification of consonant groups and omission of consonants [49]. Regarding the lexicon, comprehension at an early age may be similar to that of typically developing children (TD) of similar ages, but expressive vocabulary is significantly lower [50,51]. At the morphosyntactic level, the mean length of verbal sentences is lower than that of children with typical development, presenting difficulties with the production of grammatical morphemes and auxiliary verbs [52]. With regards to pragmatic competence, although it is lower when compared to children with typical development, it is considered a relatively strong area in DS [1]. Most research agrees that children with DS have communicative intention from an early age that they convey as much with vocalizations as with gestures. Later on, and despite structural difficulties, they are able to carry on a conversation. In addition, their discursive skills are acceptable [52,53].

Finally, the linguistic profile of developmental language disorders can vary a great deal, especially if we consider it as a severe and persistent disorder of the acquisition and development of oral language. DLD can affect one or more components of language to different degrees, both at the expressive and comprehensive levels [54]. Most studies seem to indicate that there is a pattern of impairment in aspects related to vocabulary and, particularly, in morphosyntax [55]. At an early age, fewer vocalizations and lower complexity of syllabic structures are observed. Children with DLD show difficulties in the repetition of pseudowords, which is considered one of the diagnostic markers [55,56]. Phonological awareness disorder is responsible for the fact that 80% of DLD children present difficulties in the acquisition of literacy [57]. Children with DLD also show a restricted lexicon, frequently using circumlocutions or expressions such as “thing” for multiple references, or using verbs such as “go” or “do”, embedded in different expressions, to make reference to a multitude of actions [56]. They show evocation difficulties and are slower and less precise in naming tasks, where semantic and phonological paraphasias are common [58].

Morphological and syntactic deficits are considered the main disorder in DLD. In Spanish, the most prevailing difficulties are found in the use of articles, clitics and verbs in the subjunctive, the use of plural inflection and gender agreement between determiners nouns and adjectives [55,59,60]. At the syntactic level, they have difficulties repeating sentences and tend to use simple structures and show difficulties in the understanding and using of complex syntactic structures, with mistakes in the order of elements in complex sentences [56,61]. Impairments in the use of demonstratives, possessives, prepositions and pronouns and in the understanding of pronominal anaphoras and reflexive terms are also common in children with DLD.

In relation to pragmatic skills, there is no consensus on whether the difficulties are primary or are a consequence of the impairment in the other areas of language [55]. DLD children show difficulties in taking into account the interlocutor’s needs during conversations and in adapting their language to context. It is also common that they show problems in the ability to give appropriate responses and to repair conversational breakdowns [56]. With regards to narratives, children with DLD manifest difficulties in the use of cohesion mechanisms and produce many grammatical errors [62]. On the other hand, although all pragmatic functions are present, their use is deficient [56].

Both the CCC-2 [2] and its initial version, the CCC [63], have been used in different studies to compare the linguistic profiles of different clinical groups, among which are ASD, ADHD, DS and DLD [64,65]. Most studies, however, are focused on English-speaking samples. Only a few studies have been carried out with Spanish-speaking children [12,64,66] and none with Galician-speaking children. The differences that exist in the structural and usage (pragmatic) aspects between different languages make it necessary to carry out specific studies. At the same time, although the changes in linguistic and communicative ability throughout development are important, there are very few studies that try to compare children in the early ages of development with children in later ages [65].

For this reason, this study was planned with the aim of assessing the usefulness of the Galician version of the CCC-2 [1] for the differential identification of ASD versus ADHD, DS, DLD and typically developing children (TD) in a function of the linguistic profile they show. On one hand, the capacity to discriminate disorders with a predominance of a pragmatic deficit (ADHD and ASD) from those with a predominance of a structural deficit (TDL and DS) was tested. On the other hand, we also studied the capacity to distinguish between profiles in which pragmatic impairment is the characteristic feature (ASD and ADHD). Additionally, in order to verify the usefulness of the Galician CCC-2 at different ages, the study was carried out with both early (preschool) and later age (school age) children.

## 2. Materials and Methods

### 2.1. Participants

The sample is made up of a total of 117 participants, all of them children between the ages of 4 and 16, who present different conditions: ASD, ADHD, DLD, DS and typical development (TD). Information about their communication skills was provided by the parents.

Regarding the inclusion criteria, all the participants had to present oral language and combine at least two words. Furthermore, Galician should be the dominant language in their environment. In the case of preschool children in the clinical groups, given their young age and the impossibility of being sure of certain diagnoses up to school age, the children were included with a diagnosis supported by neuropediatricians on the basis of previous evaluations by speech therapists and/or psychologists. Regarding the exclusion criteria, they should not have hearing loss or comorbid disorders and their IQ should be within normal limits (with the exception of the group with DS because low IQ is a trait associated with this profile).

The participants who fulfilled the aforementioned criteria were distributed into 10 groups, taking into account two variables: the clinical condition (diagnosis of ASD, ADHD, DLD, DS or TD) and age. The participants of each clinical condition were divided into two age groups, 4–6 years and 7–16 years, establishing the cut-off point in the age limit to receive early attention services. These groups are comparable in gender (χ^2^ = 2.76; df = 9; *p* = 0.97) and no difference was observed in the mean age among the 5 groups of preschool children (F = 2.23; df = 4.56; *p* = 0.08) nor of school age children (F = 0.98; df = 4.51; *p* = 0.43).

The informants were mostly mothers, with no differences between the groups regarding the gender of the informants (χ^2^ = 7.39; df = 9; *p* = 0.60) or their educational level (χ^2^ = 14.28; df = 27; *p* = 0.98). The sociodemographic characteristics of the participants and the informants can be seen in Table 1.

### 2.2. Instruments

The instrument used is the Galician version of the Children’s Communication Checklist—2nd edition, which is an adaptation of the items of the original version [2] prepared by Carril and Pérez Pereira [1].

The CCC-2 consists of 70 items, distributed in 10 scales (see Table 2). The A-D scales assess the structural aspects of language and the E-H scales pragmatic aspects. The last two (I, J) collect information on social communication. The answers, provided by the parents, teachers or anyone who had had frequent contact with the children were collected on a four-point Likert-type scale, based on the frequency of occurrence of the behaviors.

From the above-mentioned scales, a general communication index (GCI) can be calculated, based on the sum of the first eight scales (A to H). This allows for the identification of subjects with clinically significant communication problems. Since there are no standardized scales for the Galician version (there are no scalar scores) and direct scores were used, high values indicated higher levels of impairment. On the other hand, a social interaction deficit index ((SIDI) ((E + H + I + J) − (A + B + C + D)) was calculated, which indicates to what extent the sociopragmatic difficulties are higher or not in relation to the structural ones. In this case, a negative score indicates that the structural problems (the sum of A + B + C + D) are higher that the sociopragmatic ones (the sum of E + H + I + J) and a high positive score indicates that the sociopragmatic problems are higher than the structural ones. In our case, in addition to these indices and following studies such as the one by Philofsky et al. [67], we calculated a general pragmatic index (GPI) (E + F + G + H), a general structural index (GSI) (A + B + C + D) and a social communication index (SCI) (I + J).

### 2.3. Procedure

A cross-sectional survey methodology was used, based on the individual application of the Galician version of the CCC-2. In all cases, the parents were asked for informed consent and sociodemographic information. The research was approved by the Ethics Committee of the UDC (University of A Coruña).

The questionnaires were administered online, using the “Google Forms” application. In the case of children with TD, the parents were contacted mainly through the telephone messaging service (direct contact and dissemination in school groups of mothers and fathers), explaining the object of the study and requesting their collaboration by answering the questionnaires through the attached links. Regarding the clinical sample, contact with the parents was made mainly through a public early intervention service and different private clinics, associations and organizations of parents in public schools (AMPA). In this way, a total of five associations, six private services and one AMPA collaborated. The study was also disseminated through social networks for a greater reach.

The parents filled out the CCC-2 in about 15–20 min. The scoring took about 5 min per report.

### 2.4. Analyses

To analyze the data obtained, the SPSS statistical package was used (version 26.0 for Windows). First, the internal consistency of the Galician CCC-2 was analyzed through Cronbach’s alpha coefficient. In addition, the ability of the instrument to classify the subjects into the different clinical groups and the control group has been checked by means of a discriminant analysis.

Descriptive statistics were calculated for the two age groups with TD in the different scales and indices and for the clinical groups. After verifying that the groups were comparable in different variables through the ANOVA and Pearson’s Chi-square tests, comparisons were established between them using the Kruskal–Wallis test. Thus, the five groups of preschoolers created (4–6 years of age) based on their clinical condition (ASD, ADHD, DLD, DS and TD) were compared to each other. The same procedure was carried out with the five groups of school age children (7–16 years of age). Subsequently, the effect size was calculated and post-hoc analyses (Bonferroni adjustment) were performed to establish comparisons between pairs of groups. Finally, the two age ranges (preschool and school age) in each clinical group and the control group were compared using the Mann–Whitney U test.

## 3. Results

The study of the reliability of the Galician adaptation of the CCC-2 through the analysis of internal consistency revealed a Cronbach’s alpha of 0.91 for the whole sample. The internal consistency of each scale can be seen in Table 3.

The discriminant analyses carried out with the composite scores general communication index and social interaction deficit index revealed a sensitivity of 97.83% and a specificity of 93.33% in the case of children aged 4–6 years and a sensitivity of 97.56% and a specificity of 100% in the case of school age children 7–16 years. Likewise, the Galician version of the CCC-2 allows for discrimination between clinical profiles, correctly classifying 82.61% of preschool children and 90.24% of school age children into clinical groups. In the youngest age group, the classification errors are registered mainly between the groups with DLD and DS, with an analysis of the different scales being necessary for an adequate classification in these cases.

Furthermore, given the absence of scalar scores or reference scales differentiated for Galician preschoolers and school age children, the mean and standard deviation of the direct scores obtained in the groups with TD children were calculated for comparison purposes.

Regarding the differences between the linguistic profiles of the different groups, Table 4 shows the comparative analyses carried out using the Kruskal–Wallis test at the preschool age, which indicate significant differences between the five groups in all scales and indices.

In a similar fashion, Table 5 shows the comparative analyses carried out using the Kruskal–Wallis test at school age, which also indicate significant differences between the five groups in all scales and indices.

In general terms, TD children, who are used as reference, show good linguistic development (low scores on the general communication index), although the group of TD preschoolers shows the most impaired results (higher scores) compared to that of school age children. Parallel results can be appreciated in all the linguistic scales (A to H) and the general pragmatic and general structural indices. The positive social interaction deficit index values obtained by the TD children show that the pragmatic and social interaction difficulties (scales E + H + I + J) were slightly higher than the structural ones (A + B + C + D) in both age groups, although the difference was slight.

The results of the Kruskal–Wallis tests indicate that there were significant differences between groups in all the CCC-2 indices and in all the individual scales (A to J) in both preschool (Table 4) and school age children (Table 5).

Comparisons between pairs of groups were established to identify those differences, which are responsible for the results found. Post-hoc analyses (Bonferroni adjustment) were used with *p* < 0.05. Table 6 shows the post-hoc analyses, which indicate between which groups there were significant differences in each of the scales and indices.

As Table 6 shows, typically developing children scored the lowest on virtually all scales at the preschool and school age, as expected. These results indicate that their performances were significantly better than those of the clinical groups. There are always significant differences between the TD group and any other clinical group, although the precise group varies depending on the measures. The only exception to this general result is the score obtained in the social interaction deficit index, in which the DS and DLD groups obtained the lowest results at preschool and school age.

At the preschool age, all the clinical groups differ from the group with TD in the four structural scales (A, B, C and D), with the exception of the ADHD group, which obtained relatively better results (lower scores) than the other clinical groups. The differences between the scores of the ADHD group and those of the ASD, DLD and DS groups reach significance in scales A (speech) and B (syntax) and there is also a significant difference between the ADHD group and the DLD in scale D (semantics).

In relation to the scales referring to pragmatic aspects, preschoolers with ASD show significantly higher scores (higher disorders) with respect to the TD group in all of them (E, F, G and H), obtaining the highest results of all the clinical groups. Thus, there were also significant differences between the ASD group and the DS and DLD groups in scales E (inappropriate initiation), F (stereotyped language) and H (non-verbal communication), between the ASD and the ADHD group in the F and G (use of context) scales and between the ASD and the DLD group in the G scale as well. The ADHD group also shows very high scores in several pragmatic scales, which results in significant differences with the TD group in the scales E (inappropriate initiation), F (stereotyped language) and H (non-verbal communication). The DS group also shows significant differences with the TD group in the G (use of context) scale and the DLD group with the TD group in the F and G scales, indicating that some pragmatic abilities of DS and DLD children may also be impaired.

In relation to the social communication scales (I and J), the ASD shows the highest results and this means significant differences with all the other groups (TD, ADHD, DLD and DS) in scale I (social relations) and with the TD, DLD and DS groups in scale J (interests). The scores of the DLD group were also significantly higher than those of the TD group in the I (social relations) scale.

**Figure 1:**
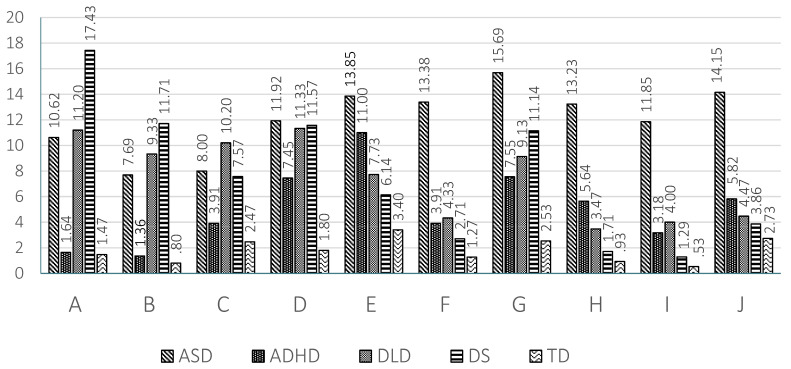
Means obtained by the 5 groups at preschool age in the 10 scales of the Galician CCC-2.

**Figure 2:**
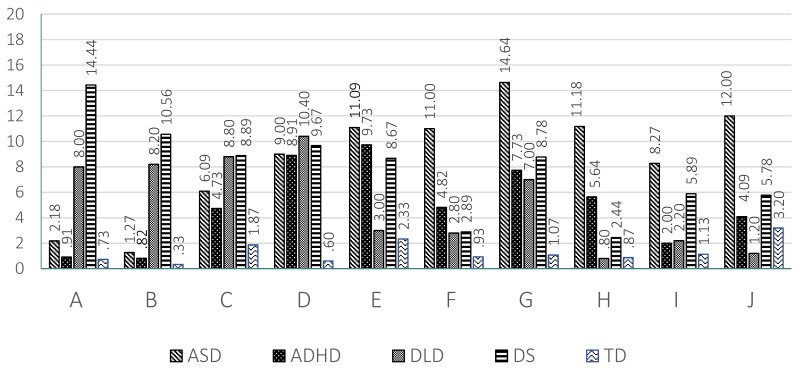
Means obtained by the 5 groups at school age in the 10 scales of the Galician CCC-2.

The results found in the 10 scales are graphically represented in Figure 1 for preschool children and in Figure 2 for school age children.

The general communication index was significantly lower in the TD preschool children than in the all the clinical groups (ADHD, DLD, DS and ASD groups). A significant different in the GCI was also found between the ADHD and the ASD groups, with the ASD group obtaining the highest level of impairment (higher scores).

The social interaction deficit index indicates that the DLD and the DS groups obtained the lowest results (negative values), which reached significance when compared to the TD, ADHD and ASD groups.

In the general pragmatic index, the children with ASD obtained the highest results (higher impairment) and the comparisons with TD, DLD and DS groups shed significant differences. Significant differences also existed between the DLD and ADHD groups and the TD group.

In relation to the general structural index, the ASD, DLD and DS groups obtained significantly higher results (higher impairment) than the TD and ADHD groups.

Finally, in the social communication index, the ASD preschool groups scored significantly higher than the TD, DLD and DS groups. The ADHD group also scored significantly higher than the TD group.

At school age, the children with DLD and DS obtained significantly higher results (higher disorder) in all the structural scales (A to D) as compared to the TD children. In addition, significant differences between these groups (DLD and DS) and the ADHD group were found in scales A (speech) and B (syntax). The ASD group obtained significantly higher results that the TD group in scales C (semantics) and D (coherence) and the ADHD group obtained higher scores than the TD group in scale D.

In relation to the scales referring to pragmatic aspects, school age children with ASD obtained significantly higher scores (higher impairment) than the TD children and the DLD group in all these scales (E, F, G and H). The ASD group also shows significantly higher scores than the DS group in scales F (stereotyped language), G (use of context) and H (non-verbal communication) and higher scores than the ADHD group in scale G (use of context). For their part, the children with ADHD obtained higher scores than the TD children in all the pragmatic scales (E to H) and significantly higher scores that the children with DLD in scale H (non-verbal communication). The children with DS obtained significantly higher scores than the TD children in scales E (inappropriate initiation) and G (use of context).

In relation to the social communication scales (I and J), the school age children with ASD show the highest results. There were significant differences with all the other groups (TD, ADHD and DLD) except the DS group in scale I (social relations) and with the TD, DLD and ADHD groups in scale J (interests). The scores of the DS group were also significantly higher than those of the TD group in scale I (social relations) and significantly higher than those of the DLD group in scale J (interests).

The results of the general communication index at school age parallels those found for preschool children: all the clinical groups (ASD, ADHD, DLD and DS) obtained significantly higher scores than those of the TD children. The only difference was that at school age there was no significant difference between the ADHD and the ASD groups.

The results of the social interaction deficit index indicate that the children with DLD and DS obtained the lowest results (negative values) and the ASD and the ADHD groups the highest. Significant differences existed between the DLD and DS groups, on the one hand, and the ASD and ADHD groups, on the other. Significant differences were also found between the DLD and ASD groups and the TD group.

In the general pragmatic index, the children with ASD obtained the highest results (higher impairment) and the comparisons with the TD and DLD groups pointed to significant differences between these groups and the children with ASD. Significant differences also existed between the ADHD and DS groups and the TD group.

In relation to the general structural index, significant differences were found between the TD group (with the lowest scores) and the DLD, DS and ASD groups. There were also significant differences between the DS group (with the highest scores) and the ADHD and ASD groups.

Finally, in the social communication index at school age, there were significant differences between the ASD and DS groups (with the highest scores) and the TD group and between the ASD, DS and ADHD groups and the DLD group (with the lowest score).

**Figure 3:**
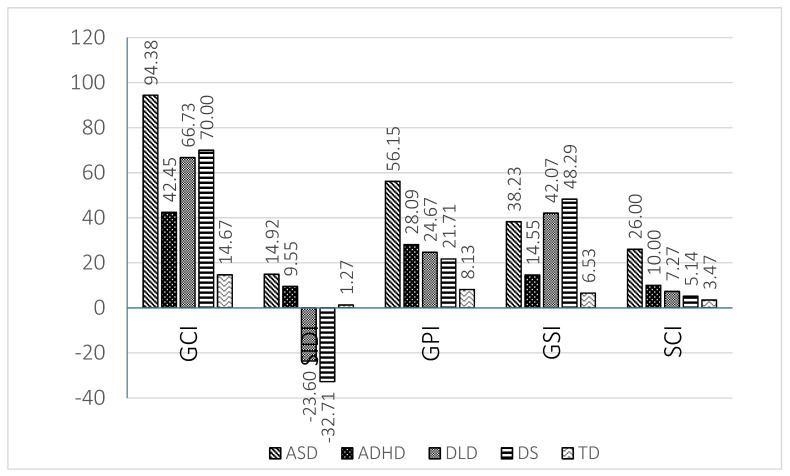
Means obtained by the 5 groups at preschool age in the indices of the Galician CCC-2.

**Figure 4:**
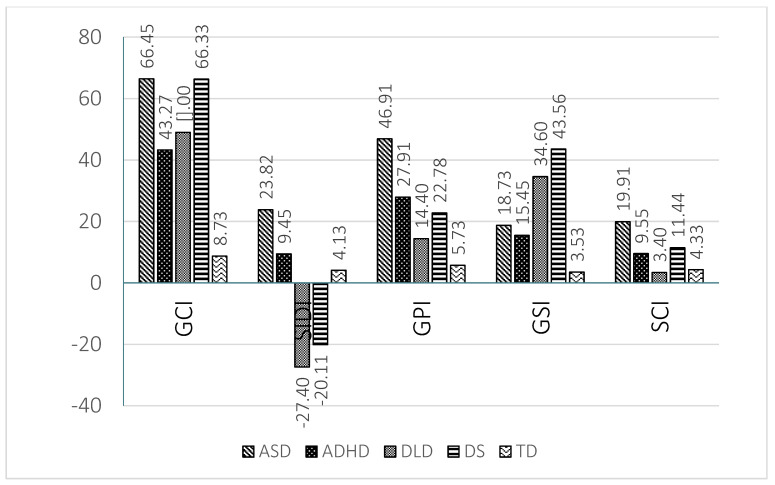
Means obtained by the 5 groups at school age in the indices of the Galician CCC-2.

The results found in the indices are graphically represented in Figure 3 for preschool children and in Figure 4 for school age children.

Finally, the comparisons between the scores of the preschool and the school age children in each group are presented in Table 7.

The results of the Mann–Whitney U test indicate a few significant differences. The most important ones revealed that children with ASD obtained better results at school age than at preschool age in scales A (speech) and B (syntax) and also (although with a lower level of significance) in scales D (coherence), F (stereotyped language) and I (interests). For their part, children with DLD obtained significantly better results (that is, lower scores) at school age than at preschool age in scales E (inappropriate initiation), H (non-verbal communication) and J (social relations). TD children show moderately better results at school age than at preschool age in scale G (use of context). In contrast, children with DS obtained a higher level of impairment (higher scores) at school age when compared with those of preschool age in scales E (inappropriate initiation) and I (interests) and children with ADHD also obtained higher scores (higher impairment) at school age than at preschool age in scale D (coherence).

The results obtained in the general communication index point to a better performance (lower scores) of children with ASD and DLD at school age than at preschool age.

In relation to the social interaction deficit index, the children with TD, ASD and DS show higher scores at school age than at preschool age.

The children with ASD and DLD show significantly lower scores (lower impairment) in the general pragmatic index at school age than at preschool age. The same trend can be observed for the children with ASD in the general structural index and the social communication index. In contrast, the children with DS obtained a significantly higher level of disorder (higher scores) at school age than at preschool age in the social communication index.

## 4. Discussion

The Galician version of the CCC-2 for preschoolers and school age children shows high internal consistency, with values similar to those previously found by [1] and those reported in other languages [2,12,68].

Regarding the reference scores of children with TD in the general communicative index, they revealed a low level of language difficulties as might be expected. The same idea can be drawn from the comparison of the results obtained by the TD children with those of the clinical groups in the different scales and complex indices. In all cases the TD children obtained significantly better results than the groups of children with neurodevelopmental disorders, with the exception of SIDI. This may indicate that the main difficulties in the TD group affect sociopragmatic aspects (positive SIDI), which coincides with the results found by different authors [12,65,69,70,71]. The difference found between the sociopragmatic and the structural scores (SIDI) was greater in school age children compared to preschool age children, as the U test shows, probably due to the persistence of these difficulties at later ages.

Regarding the linguistic profiles of the different clinical groups, in ASD a predominance of pragmatic and social communication disorders is observed. The ASD group obtained the highest scores, which indicate serious impairment, in the four pragmatic scales: inappropriate initiation (E), stereotyped language (F), use of context (G) and non-verbal communication (H) (see Figure 1 and Figure 2). Accordingly, the GPI of the ASD group shows the greatest impairment in pragmatics at both ages. In the same vein, the children with ASD show the highest scores in the two scales related to social communication: social relations (I) and interests (J). These results coincide with those found by other authors [1,2]. Logically, the social communication index of the ASD group at school age and preschool age revealed an important level of impairment. Therefore, the ASD group shows the highest impairment in all the pragmatic and sociocommunicative scales at preschool and at school age (see Figure 1 and Figure 2). The relatively most intact areas for these children relate to the formal aspects of language, especially speech (A) and syntax (B) at school age, but not at preschool age, supporting the findings of other studies [65,71,72]. The significant difference found in the U test between preschool and school age children in the scores of scales A (speech), B (syntax) and D (coherence) might, tentatively, reflect developmental improvements in the structural aspects of language in children with ASD. Something similar is also reflected in the significant difference found between preschool and school age children with ASD in the general communication index. The relatively higher performance in the structural dimensions of language in school age children with ASD is also reflected in the SIDI score, which is higher in school age than in preschool age children, as was reported in another study [73]. Therefore, the results of our study point to differences between preschool and school age children with ASD. While at preschool ages children with ASD differed from TD children on all the structural scales, coinciding with results found by another study [65], at school age the differences between ASD and TD groups in speech and syntax disappear [10,12,71] probably due to improvement in these areas (which should be studied in a longitudinal research).

The ADHD group presents a similar profile to that of the ASD group, although with a lower level of severity. The areas of greatest impairment were those scales related to pragmatic abilities (E to H). The ADHD group showed significantly higher scores than the TD children in the scales of inappropriate initiation (E), stereotyped language (F), use of context (G) and non-verbal communication (H) at preschool and school ages (see Figure 1 and Figure 2). Logically the GPI was also impaired. The impairment in scale H was significantly higher than in the DLD group, as well. In contrast, in the scores of scales A (speech) and B (syntax) there was no significant difference with TD children at preschool and school age. This result indicates a relatively preserved capacity for speech (phonology) and syntax in the children with ADHD. In scale C (semantic) there was a significantly higher score than that of TD children at preschool age, but not at school age. The contrary occurs in scale D (coherence). In children with ADHD difficulties increased in several scales at school age compared to preschool age; this increase reached significance in scale D (coherence). This trend was reflected in the score obtained in the general structural index, which was significantly higher than that of TD children at school age but not at preschool age. The difficulties of the ADHD group became more evident as cognitive and linguistic demands increased. Other studies [65,71] also report the absence of structural deficits in Dutch and Norwegian school age children with ADHD, except for coherence, as was found in the present study. To our knowledge, there are no studies with CCC-2 at preschool age in the ADHD population that allow us to compare the results. The scores obtained by the ADHD group in the social communication index were significantly higher than those of the TD group, but significantly lower than those of the ASD group both at preschool and school age. The same result is clearly observed in scale I (social relations) at both ages, although the difference was only significant between the ADHD group and the ASD group at school age. Therefore, the social communication index and scales I and J (to a lesser extent) permitted a differential diagnosis between children with ADHD and with ASD. The children with ADHD show high scores in the social interaction deficit index, the difference being significant in relation to DLD and DS children at both ages. The SIDI, however, permitted differentiation between the children with ADHD and with ASD, the scores of the latter being higher (see Figure 3 and Figure 4).

With regards to DLD a clearly differentiated profile can be also observed. A predominance of structural impairment is observed at both school and preschool ages. The high scores obtained by the DLD group in the structural scales (A to D) stand out and point to severe impairment in this area (see Figure 1 and Figure 2). The difference reached significance when compared to the TD and ADHD groups in scales A (speech) and B (syntax) and when compared with the TD group in scales C (semantics) and D (coherence) at both ages. The scores of the DLD group, however, were higher at preschool age than at school age. The high scores obtained in the general structural index, which reached significance when compared to TD children, endorsed the impairment of the children with DLD in the structural dimensions of language. There were also pragmatic deficits, given their interrelation of pragmatics with the formal aspects of language [74]. Disorders in the use of context (G) stand out, especially in the understanding of idiomatic phrases, irony and jokes. In general terms the DLD children did not have significantly higher scores than those of TD children in the four pragmatic scales (E to H), with the exception of scales F and G at preschool age. Children with DLD had significantly lower results than those with ASD in scales E, F, G and H, which permitted a clear differentiation between these two groups. In addition, in preschool children a greater level of inappropriate initiation (E) of conversations is observed, with difficulties decreasing at school age [2,75]. There appeared to be significant differences between DLD preschool and school groups in scales E (inappropriate initiation), H (non-verbal communication) and J (interests), which could point to a clear trend of progress. This may respond to advances in formal language skills and memory capacity [55]. In preschool children with DLD deficits in the use of context (G) and social relations (I) also appeared, although these differences disappeared at school age, which is indicative of the importance of oral language in interaction with peers [55] and of how children with DLD are able to progress. This difference is clearly observed if we compare the scores in the general communicative index at preschool and at school age. Finally, the children with DLD characteristically show very low scores with negative values in the SIDI, as Figure 3 and Figure 4 show.

Concerning the DS group profile, there was a predominance of deficits in all the structural scales: speech (A) syntax (B), (C) semantics and (D) coherence in both age groups (see Figure 1 and Figure 2). Consequently, the scores obtained in the GSI were also significantly higher than those of TD children at school age and higher than those of the TD and ADHD groups at preschool age. Children with DS, however, also show difficulties in the pragmatic areas, particularly in scales E (inappropriate initiation), F (stereotyped language) and G (use of context). In most of the pragmatic scales children with DS had significantly lower scores than children with ASD at preschool and school age. Pragmatic problems were also reflected in the relatively high scores observed in the GPI at both ages. In the school age DS group, there was also an increase in the level of impairment in the scales of social relationships (I) and J (interests). Children with DS show a high impairment level in different areas and not only in the structural ones. Comparing the performance of preschool and school age children with DS a shorter difference can be appreciated than that observed in the other clinical groups, with school age children showing significantly higher results (higher impairment) than preschool children in scales E (inappropriate initiation) and I (social relations) and in the SIDI and SCI indices. The same as with ADHD there was a relative increase in difficulties in some areas of linguistic development between the preschool and the school age groups, although a larger number of areas were affected, apart from those already commented on at the level of semantics (C) and social relations (I). This could be explained again by an increase in the complexity of the demands in these areas and a slower development in children with DS. Although interaction is generally considered to be a strong point of people with DS, social relationships are very complex and require good linguistic and cognitive development, aspects in which DS children present difficulties, giving rise to poor action schemes and differences in interests [76]. Coinciding with our study, Smith, Næss and Harrold [77] find differences in DS preschoolers when compared to the TD group in the pragmatic scales, which might be a reflection of the linguistic and cognitive problems that children with DS have. Typically, children with DS show a very low score in SIDI at preschool and school age, obtaining negative values, which indicates their serious difficulties with the structural aspects of language.

In addition to the capacity for identifying the linguistic profile of different disorders, the CCC-2 was effective in detecting language and communication difficulties in both preschool and school age children through the general communication index. The GCI demonstrated a capacity for discriminating those children with linguistic disorders from those that did not present them at both ages. Figure 3 and Figure 4 very clearly show how all clinical groups show difficulties in this index, particularly the ADHD and DLD groups.

In addition, the Galician CCC-2 allowed us to discriminate profiles with a predominance of structural disorders from those with a predominance of pragmatic disorders, both at school and preschool ages. The SIDI also permitted us to differentiate at both periods of age between DLD and DS, two disorders with serious impairment of structural aspects, on the one hand, and ASD and ADHD, two disorders with a predominance of pragmatic impairment on the other (see Figure 3 and Figure 4). Children with DLD and DS obtained very low results (negative values) in the SIDI index, while ADHD and particularly ASD obtained very high results. These findings reinforce those found by Bishop [2], who points out the greater capacity of the SIDI to discriminate impaired profiles.

The general pragmatic index allowed us to establish clear differences between ASD and the other neurodevelopmental disorders, particularly with children with DLD. The ASD group shows the worst results in the GPI at preschool and at school age.

The general structural index established differences between the DLD and DS groups with respect to the ADHD group.

Finally, the social communication index shows the great impairment of the children with ASD in relation to the other clinical groups, and particularly the DLD group.

In addition to the aforementioned indices, a detailed analysis of the differences in the results obtained in the 10 scales ensures a better differential diagnosis, particularly when taking into account the changes observed with age in the linguistic profiles.

Thus, although the high structural impairment in preschoolers with ASD, which can even overcome the pragmatic deficit [65], complicates differential identification with respect to DLD children, an individual analysis of the scales revealed that children with ASD show a significantly greater deficit than the DLD children in all the pragmatic scales at preschool and school age (as already commented on in the description of the profiles) coinciding with the findings of Helland [78]. Geurts and Embrechts [65] also find differences in the G scale (use of context). At school age, however, differences between the DLD and the ASD groups became more evident (for instance in scales A and B). Thus, at preschool age, the differential diagnosis between DLD and ASD should be made based on the lower pragmatic and social communication disorders of children with DLD compared to children with ASD, without paying attention to structural aspects exclusively. These are particularly useful for discriminating between these profiles at school age [75].

Regarding DLD and ADHD, the SIDI allowed us to discriminate between the two at both age ranges, given the higher level of impairment of ADHD compared to that of DLD in this index. If we take into account the individual scales, at preschool age there would be no significant differences in pragmatic or social communication skills between these children. At school age, the greater pragmatic difficulties of children with ADHD in scale (E) inappropriate initiation and (H) non-verbal communication also contribute to the differentiation from children with ADHD. Children with DLD show greater structural impairment in scales A and B at both ages and also in scale C (semantics) at preschool age. The higher level of impairment of children with DLD in several structural scales allowed us to discriminate between DLD and ADHD. Helland, Biringer, Helland and Heimann [71], however, do not find these differences in the pragmatic scales at school age. As far as we know, there are no other studies comparing both clinical groups at preschool age.

In relation to the comparison between DS and ASD, the areas that allowed us to discriminate between them varied according to age. At preschool age, the scales referring to pragmatics (scales E, F and H) and social communication (I, J), highly disordered in ASD, allowed us to differentiate between both clinical groups. At school age, the differences in pragmatics (F and H) still continued, but the differences in the areas referring to social communication (I and J) disappeared. This, probably, was a response to an increase of impairments at school age in children with DS (scales E and F) and a clear decrease of impairments in ASD (scales E and I) (see the results of the Mann–Whitney U test). If we attend to the structural scales, there would be no significant difference between DS and ASD groups at preschool age in any of these scales. Both groups show an important level of impairment in them. At school age, however, children with DS show higher levels of impairment in scales A and B than children with ASD. This is probably a reflection of the different patterns of development of children with ASD and with DS, which was clearly positive in the case of ASD and with more difficulties in DS. The comparison of the results of preschool and school age children seemed to support this hypothesis. In the case of children with DS, cognitive impairments create more difficulties for the development and a greater distance in interests in relation to their peers, generating difficulties in social relationships [75]. Similar results were obtained by Carril and Pérez-Pereira [1] with school age children. We are aware of no studies with which to make comparisons at preschool age. In addition, the differences in the scores obtained by children with ASD and DS in the social interaction deficit index allowed us to differentiate between them with clarity. Children with ASD obtained much higher results in the SIDI than children with DS at preschool and at school age.

With regard to the comparison between children with DS and ADHD, the differences clearly appeared in the greater impairment of speech (A) and syntax (B) in the DS group in both age ranges. Although in the ADHD group the pragmatic disorder predominated while it was the structural disorder in DS children, in children with DS the pragmatic disorder was similar to that of children with ADHD, because the impairments in the formal aspects of language may have a negative impact on the development of pragmatics [74]. Similar results were also found by another study [1] with school age children. Finally, the results obtained by these children in the SIDI allowed us to differentiate between them, since ADHD children obtained higher scores in this index.

The CCC-2 also shows an adequate capacity for discrimination between profiles with a predominance of pragmatic disorder. Preschoolers with ASD present greater impairment than those with ADHD in pragmatic aspects (F and G) and social communication (I). For their part, school age children with ASD show greater impairment than children with ADHD in several pragmatic (G: use of context) and social communication scales (I: social relations and J: Interests). There was also a greater impairment of speech (A) and (B) syntax in the clinical group of preschool children with ASD with respect to the ADHD group. These differences disappeared in the school age groups. The differences found between children with ASD and ADHD in the social communication scales and the SCI confirmed the usefulness of these two scales (I and J) and the social communication index for the identification of ASD. Other studies [65,66] also find differences between ASD and ADHD children in some pragmatic scales in school age children. The higher level of impairment in the SIDI that children with ASD show in comparison to that of children with ADHD can be also used to differentiate between these groups.

Regarding the differentiation of profiles with a predominance of structural impairment (DLD and DS), no significant differences were found in any of the scales in preschool children, while in school age children there were differences in scales E (inappropriate initiation) and J (interests). In these scales the DS group obtained higher scores (higher impairment). The comparison of preschool and school age children indicates that school age children with DLD tended to present significantly better results than preschool children in several scales (E and J) and indices (GCI and GPI), while school children with DS show significantly lower results than preschool children in several scales (E and I) and indices (SIDI and SCI). In general terms, children with DLD tended to show slightly lower scores (lower impairment) than children with DS in several scales and indices, particularly at school age (see Figure 3 and Figure 4).

## 5. Conclusions

The Galician version of the CCC-2 presents adequate reliability (internal consistency) to assess language and communication in preschool and school age children. This tool allows for a screening of Galician children with language difficulties in a wide age range (4–16 years). Furthermore, it enabled the differential identification of disorders in which there was a predominance of the pragmatic deficit (ASD and ADHD) compared to those in which structural alteration predominated (DLD and DS). Although its global indices (GCI and SIDI) did not allow for discriminating between disorders characterized by a pragmatic deficit, the information provided by the different scales made it possible to identify the profile of ASD versus ADHD. Likewise, the detailed analysis of the linguistic profiles and their different development with age made it possible to discriminate between profiles with a predominance of structural impairment (DLD and DS). This instrument also allowed us to appreciate the development of the linguistic profiles of different disorders, providing relevant information for the differential diagnosis at different developmental stages. The knowledge of the specific profiles at an early age, which are not studied much so far, makes it possible to identify the initial warning signs in each disorder, which can be confirmed in later developmental stages.

In this way, a screening tool adapted to our language and context is provided, which can be used both in early intervention services and by those that work with school age children. The CCC-2 permits the simultaneous screening of different pathologies, provides information on different areas of language and helps in monitoring the effects of intervention.

Despite the potential usefulness of the results found, this study had a series of limitations that must be taken into consideration for future research. Some of these limitations are related with the sample and its selection process, since it was of limited size and lacked representativeness. Therefore, the study should be considered as a pilot study. Another limitation is related to the need for a normative adaptation of the Galician CCC-2 and the establishment of scales with scalar scores. Finally, it would be interesting to incorporate the analysis of new clinical groups in future studies, such as the social communication disorder, so that the instrument could improve its diagnostic capacity. Longitudinal studies would also be necessary to investigate the process of developmental change in these neurodevelopmental disorders.

## Figures and Tables

**Table 1:** Sociodemographic characteristics of the children and their informants.

		N	Participants			Parents						
			Age	Gender		Age	Gender		Educational Level			
				Female	Male		Female	Male	Primary	Secondary	High School and Technical	University
			M (SD)	%	%	M (SD)	%	%	%	%	%	%
**Age Group 4–6**	TD	15	5.40 (0.63)	33.30	66.70	35.87 (4.61)	86.70	13.30	20.00	26.70	20.00	33.30
	ASD	13	4.85 (0.80)	30.80	69.20	36.00 (4.08)	92.30	7.70	23.10	30.80	15.40	30.80
	ADHD	11	5.36 (0.67)	36.40	63.60	37.36 (4.65)	100	0	27.30	9.10	45.50	18.20
	DLD	15	4.93 (0.80)	33.30	66.70	36.20 (2.70)	86.70	13.30	6.70	20.00	40.00	33.30
	DS	7	5.57 (0.54)	28.60	71.40	38.71 (1.98)	85.70	14.30	0	28.60	42.90	28.60
**Age Group 7–16**	TD	15	10.87 (2.62)	46.70	53.30	42.33 (6.04)	100	0	13.30	20.00	33.30	33.30
	ASD	11	12.27 (3.26)	45.5	54.5	40.55 (4.39)	81.80	18.20	9.10	18.20	18.20	54.50
	ADHD	11	11.55 (2.73)	36.40	63.60	40.45 (3.88)	100	0	9.10	18.20	45.50	27.30
	DLD	10	10.10 (2.92)	40.00	60.00	40.20 (2.78)	90.00	10.00	10.00	20.00	40.00	30.00
	DS	9	12.11 (3.37)	55.60	44.40	42.33 (5.83)	100	0	11.10	33.30	33.30	22.20

**Table 2:** Scales of the CCC-2 (adapted from Philofsky et al. [67]).

Scale	Aspects Assessed
A- Speech	Phonology, articulation and intelligibility of speech.
B- Syntax	Structure, complexity of sentences and grammatical markings.
C- Semantics	Fluency and precision in word recall and lexical access.
D- Coherence	Reference settings.
E- Inappropriate initiation	Excessive talking, persistence in initiating the same topic and not initiating topics of mutual interest.
F- Stereotyped language	Excessive use of phrases learned without adaptation to the context and excess precision.
G- Use of context	Understanding and use of social communication rules, sarcasm, figurative language, politeness, metaphors, humor and the intentions of others.
H- Non-verbal communication	Comprehension and use of non-verbal conversational cues, including gestures and facial expression.
I- Social relations	Interest in and establishment of relationships with peers.
J- Interests	Restricted and/or repetitive interests and flexibility.

**Table 3:** Reliability of the scales of the Galician CCC-2.

Scales	A	B	C	D	E	F	G	H	I	J	Total
Cronbach’s alpha	0.94	0.87	0.77	0.87	0.81	0.83	0.85	0.88	0.83	0.84	0.91

**Table 4:** Means (M), standard deviations (SD) and average ranges of the five groups at preschool age and analysis of the differences through the Kruskal–Wallis test.

Scales and Indices	TD			ASD			ADHD			DLD			DS			H	ɳ^2^H
	M	SD	Average Range	M	SD	Average Range	M	SD	Average Range	M	SD	Average Range	M	SD	Average Range		
A	1.47	2.39	13.17	10.62	4.57	39.88	1.64	1.21	16.18	11.20	5.62	41.07	17.43	2.15	54.43	43.44 ***	0.67
B	0.80	1.01	11.97	7.69	3.04	39.35	1.36	1.12	16.27	9.33	4.15	43.70	11.71	1.50	52.21	45.86 ***	0.71
C	2.47	1.77	12.83	8.00	3.76	38.77	3.91	1.70	20.41	10.20	3.88	46.37	7.57	0.98	39.21	35.10 ***	0.52
D	1.80	2.08	8.47	11.92	2.99	44.15	7.45	1.86	24.95	11.33	3.87	41.00	11.57	2.94	42.93	40.73 ***	0.62
E	3.40	2.69	12.40	13.85	2.61	50.92	11.00	3.19	40.77	7.73	4.10	28.47	6.14	1.35	23.93	37.83 ***	0.57
F	1.27	1.03	12.00	13.38	2.60	54.88	3.91	1.30	32.00	4.33	2.64	32.23	2.71	1.25	23.14	42.75 ***	0.66
G	2.53	2.00	9.17	15.69	3.17	52.38	7.55	1.57	27.50	9.13	4.26	32.60	11.14	2.34	40.14	44.18 ***	0.68
H	0.93	1.10	13.57	13.23	3.96	53.69	5.64	1.57	39.55	3.47	2.92	27.97	1.71	1.50	19.29	42.22 ***	0.65
I	0.53	0.83	13.13	11.85	2.64	54.38	3.18	2.44	30.05	4.00	2.83	34.80	1.29	1.70	19.21	42.41 ***	0.65
J	2.73	1.87	18.00	14.15	2.82	54.31	5.82	2.56	34.82	4.47	3.72	24.70	3.86	1.68	23.07	34.65 ***	0.51
GCI	14.67	10.03	8.30	94.38	19.62	51.54	42.45	7.00	23.32	66.73	25.94	37.23	70.00	4.04	40.21	47.74 ***	0.75
SIDI	1.27	5.99	32.07	14.92	8.12	50.69	9.55	5.03	45.18	−23.60	11.21	13.40	−32.71	8.86	7.57	50.08 ***	0.79
GPI	8.13	4.69	9.03	56.15	9.15	54.65	28.09	5.15	36.68	24.67	12.08	29.87	21.71	3.82	27.64	47.56 ***	0.74
GSI	6.53	6.37	9.97	38.23	11.39	40.85	14.55	3.91	19.18	42.07	15.44	43.27	48.29	6.63	50.07	45.21 ***	0.70
SCI	3.47	2.50	15.20	26.00	3.29	54.42	10.00	3.32	37.59	7.27	6.19	26.50	5.14	2.80	20.64	39.59 ***	0.60

*** *p* < 0.001.

**Table 5:** Means (M), standard deviations (SD) and average ranges of the five groups at school age and analysis of the differences through the Kruskal–Wallis test.

Scales and Indices	TD			ASD			ADHD			DLD			DS			H	ɳ^2^H
	M	SD	Average Range	M	SD	Average Range	M	SD	Average Range	M	SD	Average Range	M	SD	Average Range		
A	0.73	0.88	16.90	2.18	2.32	24.27	0.91	1.30	17.14	8.00	2.75	42.50	14.44	4.07	51.33	39.95 ***	0.67
B	0.33	0.72	14.63	1.27	1.27	23.09	0.82	0.75	20.86	8.20	1.93	45.00	10.56	3.21	49.22	41.64 ***	0.70
C	1.87	0.64	9.80	6.09	2.98	31.55	4.73	1.85	26.00	8.80	3.23	42.30	8.89	2.21	43.67	35.84 ***	0.59
D	0.60	0.91	8.70	9.00	3.10	33.77	8.91	3.45	34.77	10.40	3.31	37.90	9.67	2.96	36.94	30.98 ***	0.49
E	2.33	1.95	12.67	11.09	3.70	43.18	9.73	3.00	40.27	3.00	2.67	15.00	8.67	2.50	37.56	38.66 ***	0.64
F	0.93	1.49	13.03	11.00	1.90	50.73	4.82	2.75	32.86	2.80	1.69	25.40	2.89	1.97	25.22	36.07 ***	0.59
G	1.07	1.28	9.17	14.64	2.16	50.05	7.73	3.55	30.59	7.00	3.62	27.55	8.78	1.72	32.89	41.52 ***	0.70
H	0.87	1.30	14.60	11.18	3.34	49.91	5.64	1.96	39.59	0.80	0.79	15.10	2.44	1.51	26.83	42.64 ***	0.72
I	1.13	1.46	17.43	8.27	3.13	46.73	2.00	1.90	22.64	2.20	3.01	20.40	5.89	2.42	40.83	30.60 ***	0.48
J	3.20	1.27	22.27	12.00	4.27	49.91	4.09	2.34	27.77	1.20	0.79	8.80	5.78	2.73	35.50	38.18 ***	0.63
GCI	8.73	3.63	8.07	66.45	12.36	43.59	43.27	11.61	26.36	49.00	17.71	30.75	66.33	8.19	44.22	41.76 ***	0.70
SIDI	4.13	3.72	28.77	23.82	10.60	49.86	9.45	3.78	38.73	−27.40	6.48	7.60	−20.11	10.88	12.67	48.16 ***	0.83
GPI	5.73	3.73	9.27	46.91	7.70	51.00	27.91	7.05	36.64	14.40	7.38	21.20	22.78	3.56	31.22	46.87 ***	0.80
GSI	3.53	1.25	9.00	18.73	7.79	28.77	15.45	4.91	24.00	34.60	10.58	43.25	43.56	8.03	49.78	46.26 ***	0.79
SCI	4.33	1.72	16.17	19.91	5.91	50.14	9.55	3.08	32.32	3.40	2.72	12.10	11.44	4.50	36.17	40.83 ***	0.68

*** *p* < 0.001.

**Table 6:** Post-hoc analysis: comparison between the groups of children with TD, ASD, ADHD, DLD and DS in the 10 scales and 5 indices of the Galician CCC-2 at preschool and school age.

Scales and Indices	Preschool Age (4–6 Years)	School Age (7–16 Years)
A	TD, ADHD < ASD, DLD, DS	TD, ADHD, ASD < DLD, DS
B	TD, ADHD < ASD, DLD, DS	TD, ADHD, ASD < DLD, DS
C	TD < DS, ASD, DLD ADHD < DLD	TD < ASD, DLD, DS
D	TD < DLD, DS, ASD	TD < ADHD, ASD, DS, DLD
E	TD < ADHD, ASD DS, DLD < ASD	TD, DLD < ADHD, ASD, DS
F	TD < DLD, ADHD TD, DS, ADHD, DLD < ASD	TD < ADHD, ASD DS, DLD < ASD
G	TD < DLD, DS, ASD ADHD, DLD < ASD	TD < ADHD, DS, ASD DLD, ADHD < ASD
H	TD < ADHD, ASD DS, DLD < ASD	TD < ADHD, ASD DS, DLD < ASD; DLD < ADHD
I	TD < DLD, ASD DS, ADHD, DLD < ASD	TD < DS, ASD ADHD, DLD < ASD
J	TD, DS, DLD < ASD	TD, ADHD, DLD < ASD DLD < DS
GCI	TD < ADHD, DLD, DS, ASD ADHD < ASD	TD < ADHD, DLD, DS, ASD
SIDI	DLD, DS < TD, ADHD, ASD	DLD, DS < ADHD, ASD TD < DLD, ASD
GPI	TD < ADHD, ASD, DLD DLD, DS < ASD	TD < ADHD, ASD, DS DLD < ASD
GSI	TD, ADHD < ASD, DLD, DS	TD < DLD, DS, ASD ADHD, ASD < DS
SCI	TD < ASD, ADHD DS, DLD < ASD	TD < ASD, DS DLD < ASD, ADHD, DS

TD: Typically developing children; ASD: Autism spectrum disorder; ADHD: Attention deficit with hyperactivity disorder; DLD: Developmental language disorder; DS: Down syndrome children; CCC-2: The Children’s Communication Checklist.

**Table 7:** Comparison between the scores obtained by preschool and school age children in each of the groups using the Mann–Whitney U statistic.

Scales and Indices	TD				ASD				ADHD				DLD				DS			
	Average Range		*U*	ɳ^2^u	Average Range		*U*	ɳ^2^u	Average Range		*U*	ɳ^2^u	Average Range		*U*	ɳ^2^u	Average Range		*U*	ɳ^2^u
Age	4–6	7–16			4–6	7–16			4–6	7–16			4–6	7–16			4–6	7–16		
A	15.60	15.40	111.00	0.49	17.42	6.68	7.50 ***	0.05	13.59	9.41	37.50	0.31	14.87	10.20	47.00	0.31	10.79	6.72	15.50	0.25
B	17.50	13.50	82.50	0.37	17.62	6.45	5.00 ***	0.03	12.91	10.09	45.00	0.37	13.60	12.10	66.00	0.44	9.36	7.83	25.50	0.40
C	16.90	14.10	91.50	0.41	14.23	10.45	49.00	0.34	10.00	13.00	77.00	0.64	14.20	11.20	57.00	0.38	7.07	9.61	41.50	0.66
D	18.23	12.77	71.50	0.32	15.31	9.18	35.00 *	0.24	8.68	14.32	91.50 *	0.76	13.80	11.80	63.00	0.42	9.86	7.44	22.00	0.35
E	17.13	13.87	88.00	0.39	14.88	9.68	40.50	0.28	13.23	9.77	41.50	0.34	16.20	8.20	27.00 **	0.18	5.36	10.94	53.50 *	0.85
F	17.73	13.27	79.00	0.35	15.58	8.86	31.50 *	0.22	10.64	12.36	70.00	0.58	14.87	10.20	47.00	0.31	7.86	9.00	36.00	0.57
G	18.73	12.27	64.00 *	0.28	13.31	11.55	61.00	0.43	10.73	12.27	69.00	0.57	14.47	10.80	53.00	0.35	11.14	6.44	13.00	0.21
H	15.97	15.03	105.50	0.47	14.69	9.91	43.00	0.30	11.36	11.64	62.00	0.51	16.13	8.30	28.00 **	0.19	6.79	9.83	43.50	0.69
I	13.83	17.17	137.50	0.61	15.69	8.73	30.00 *	0.21	13.09	9.91	43.00	0.36	15.00	10.00	45.00	0.30	4.71	11.44	58.00 **	0.92
J	14.60	16.40	126.00	0.56	14.15	10.55	50.00	0.35	13.64	9.36	37.00	0.31	16.27	8.10	26.00 **	0.11	6.29	10.22	47.00	0.75
GCI	18.67	12.33	65.00	0.29	16.81	7.41	15.50 ***	0.11	10.95	12.05	66.50	0.55	15.07	9.90	44.00 *	0.29	9.93	7.39	21.50	0.34
SIDI	12.60	18.40	156.00 *	0.69	9.54	16.00	110.00 *	0.77	10.91	12.09	67.00	0.55	14.33	11.00	55.00	0.37	5.57	10.78	52.00 *	0.83
GPI	18.33	12.67	70.00	0.31	15.65	8.77	30.50 *	0.21	11.45	11.55	61.00	0.50	15.73	8.90	34.00 *	0.23	7.86	9.00	36.00	0.57
GSI	16.83	14.17	92.50	0.41	17.04	7.14	12.50 ***	0.09	9.68	13.32	80.50	0.67	14.67	10.50	50.00	0.33	10.29	7.11	19.00	0.30
SCI	14.00	17.00	135.00	0.60	15.96	8.41	26.50 **	0.19	11.95	11.05	55.50	0.46	15.13	9.80	43.00	0.29	5.07	11.17	55.50 **	0.88

* *p* < 0.05; ** *p* < 0.01; *** *p* < 0.001.

## Data Availability

Data supporting reported results can be asked for to the authors.
